# Neural Mechanisms of Attentional Control Differentiate Trait and State Negative Affect

**DOI:** 10.3389/fpsyg.2012.00298

**Published:** 2012-08-21

**Authors:** Laura D. Crocker, Wendy Heller, Jeffrey M. Spielberg, Stacie L. Warren, Keith Bredemeier, Bradley P. Sutton, Marie T. Banich, Gregory A. Miller

**Affiliations:** ^1^Department of Psychology, University of Illinois at Urbana-ChampaignChampaign, IL, USA; ^2^School of Public Health, University of California BerkeleyBerkeley, CA, USA; ^3^Department of Bioengineering, University of Illinois at Urbana-ChampaignUrbana, IL, USA; ^4^Department of Psychology, University of Colorado at BoulderBoulder, CO, USA; ^5^Department of Psychology, University of DelawareNewark, DE, USA

**Keywords:** negative affect, attentional control, prefrontal cortex, emotion, fMRI

## Abstract

The present research examined the hypothesis that cognitive processes are modulated differentially by trait and state negative affect (NA). Brain activation associated with trait and state NA was measured by fMRI during an attentional control task, the emotion-word Stroop. Performance on the task was disrupted only by state NA. Trait NA was associated with reduced activity in several regions, including a prefrontal area that has been shown to be involved in top-down, goal-directed attentional control. In contrast, state NA was associated with increased activity in several regions, including a prefrontal region that has been shown to be involved in stimulus-driven aspects of attentional control. Results suggest that NA has a significant impact on cognition, and that state and trait NA disrupt attentional control in distinct ways.

## Introduction

Research in psychology and psychiatry is moving toward an emphasis on examining and integrating psychological and biological factors that cut across categories of psychopathology in order to identify “intermediate phenotypes” that advance our understanding of the nature and causes of psychopathology (Cuthbert and Insel, 2010; Sanislow et al., 2010). The NIMH Research Domain Criteria (RDoC) project has identified Negative Valence Systems as fundamental to the development and maintenance of psychopathology. Negative affect (NA) appears to be a dimension common to some if not all of the constructs identified thus far as key to Negative Valence Systems (e.g., fear, anxiety, loss). Efforts to isolate unique vs. overlapping factors in anxiety and depression initially targeted state NA as common to both (Clark and Watson, 1991a). Further research found that the stable personality dimension of negative temperament or neuroticism was not only common to both anxiety and depression but predictive of their onset and course (Clark et al., 1994; Ormel et al., 2004). Converging evidence has since provided an abundance of empirical support that state NA is present in most disorders and that trait NA is a risk factor for psychopathology in general (for a review, see Clark, 2005).

Although trait NA (accepted by many as essentially synonymous with negative temperament and neuroticism, see Gray and Watson, 2001) is associated with a disposition to experience negative mood states (e.g., worry, anger, fear, guilt, sadness; Costa and McCrae, 1980; Watson and Clark, 1984), it has other important correlates that persist even outside of negative mood states. Trait NA is linked to poor self-esteem, pessimism, and a negative attributional style (Clark et al., 1994; Luten et al., 1997). Further, individuals high in trait NA often dwell on failures, mistakes, and disappointments (Watson and Clark, 1984) and have deficient mood-regulation skills (Costa et al., 1986; Kokkonen and Pulkkinen, 2001). Trait NA is also associated with a greater impact of life stress on the development of affective disorders (for a review, see Ormel et al., 2004).

Cognitive dysfunction has been highlighted as another important factor in affective disorders, which are characterized by deficits in executive functions and biased cognitive processing (Mathews and MacLeod, 2005; Levin et al., 2007; Clark and Beck, 2010; Gotlib and Joormann, 2010). Trait NA is associated with biases in perception, memory, and interpretation, such that individuals with high trait NA recognize and recall negative information more readily than those with low trait NA (Martin, 1985; Larsen, 1992), interpret ambiguous information in a more negative manner (Haney, 1973), and appraise situations as more stressful and threatening (Gallagher, 1990; Hemenover and Dienstbier, 1996; Oliver and Brough, 2002). Negative mood states also influence various cognitive processes, including restricting the range of thoughts and actions that come to mind and biasing perception and memory toward negatively valenced words (Fredrickson and Branigan, 2005; Chepenik et al., 2007). In addition, both state and trait NA appear to influence judgments and beliefs (Clark and Watson, 1991b; Clore and Huntsinger, 2007).

Negative moods are associated with a bias toward a systematic processing strategy that relies more on attention to situational details than on pre-existing knowledge (for reviews, see Schwarz and Clore, 1996; Heller and Nitschke, 1997). Further, emotional states evoke emotion knowledge that is experiential and context-dependent, whereas emotional traits are associated with emotion knowledge that appears to be somewhat context-independent and based on stable beliefs about emotions in general (Robinson and Clore, 2002). Therefore, it seems likely that, in emotional contexts, certain cognitive processes are modulated differentially by transient emotional states vs. enduring traits. The present study aimed to distinguish correlates of trait and state NA, given that many studies confound their effects and preclude an understanding of potentially unique cognitive mechanisms through which they differentially contribute to symptoms of psychological disorders.

One mechanism through which trait and state NA contribute to serious psychological symptoms may be via attentional control deficits, manifested in both anxiety and depression (Derryberry and Reed, 1994; Compton, 2000; Bredemeier et al., 2011). Neuroimaging work has revealed several brain areas that appear to function abnormally in anxiety and depression during tasks involving attentional control in the context of emotional distracters, including dorsolateral prefrontal cortex (DLPFC), both dorsal and rostral portions of anterior cingulate cortex (ACC), amygdala, and parietal regions (e.g., Bruder et al., 1997; Heller et al., 2003; Engels et al., 2007, 2010; Bishop, 2008; Herrington et al., 2010). Given that anxiety and depression are both associated with dysfunction in these brain regions, it seems likely that trait and state NA are linked to disruption in some or all of these areas as well, leading to attentional control problems that may put individuals at risk for the development and maintenance of psychological disorders.

We propose that trait and state NA are associated with differential attentional control impairments, linked to dysfunction in distinct attentional networks, a top-down control system and a stimulus-driven control system (Corbetta and Shulman, 2002; Corbetta et al., 2008). Given that trait NA is associated with a somewhat context-independent reliance on beliefs and knowledge, we propose that it is associated with dysfunction in more goal-directed, top-down attentional processing, implemented by a dorsal frontoparietal network. This network is influenced by current goals, expectations, and pre-existing information. In contrast, we expect that state NA will be associated with an increase in stimulus-driven attention, implemented by a ventral frontoparietal network, given that negative moods are related to a systematic processing strategy involving attention to contextual details. The present study also integrates the theoretical and empirical foundation of these distinct attentional networks with the cascade-of-control model (Banich, 2009).

The cascade-of-control model proposes that distinct areas of DLPFC implement different functions necessary for attentional control. It posits that posterior DLPFC is involved in imposing a top-down attentional set that maintains overall task goals and biases parietal regions toward processing task-relevant information, whereas mid-DLPFC is involved in selecting and maintaining the most relevant aspects of stimuli that have been received (Banich, 2009). In support of this distinction for emotion-modulated attentional processing, Herrington et al. (2010) showed that individuals high in anhedonic depression exhibited reduced posterior DLPFC activity in response to negative stimuli. In contrast, mid-DLPFC was more active for positive stimuli, regardless of anhedonic depression levels. Herrington et al. (2010) proposed that posterior DLPFC imposes more static, persistent, context-insensitive aspects of an affective set and is associated with trait affect, whereas mid-DLPFC modulates aspects of emotion processing that are more transient, stimulus-driven, context-dependent, and related to state affect. The model also proposes a temporal cascade of processing such that DLPFC comes online first and influences later ACC activity (Banich, 2009; Silton et al., 2010). When incorrect responses are made, ACC signals back to posterior DLPFC to assert stronger top-down control on future trials (Banich, 2009).

The present study examined hypotheses based on the cascade-of-control model in the context of an emotion-word Stroop task, which requires attentional control in order to ignore distracting emotional information. Trait NA was hypothesized to be associated with decreased activation in posterior DLPFC, as well as other areas involved in top-down attentional control, in the context of emotionally arousing, distracting information. In contrast, state NA was expected to facilitate the processing of emotionally arousing, salient stimuli and thus linked to increased activation in mid-DLPFC, as well as other areas involved in the processing of contextual information. Additionally, exploratory analyses examined neural correlates of the interactive effects of trait and state NA, given that behavioral research has found that the interaction between traits and states has important implications for understanding their impact on cognition and behavior (e.g., MacLeod and Mathews, 1988; MacLeod and Rutherford, 1992; Tamir and Robinson, 2004).

Most work in the psychopathology literature investigating cognitive control deficits in emotional contexts has focused on negative stimuli. When positive stimuli are excluded, it is unclear whether the observed attentional problems are valence-specific or are driven by high levels of emotional arousal (which tend to be associated with highly valenced stimuli). There is accumulating evidence that anxious individuals selectively attend to both positive and negative stimuli (Martin et al., 1991; Becker et al., 2001; Sass et al., 2010) and that depressed individuals exhibit reduced emotional reactivity to negative and positive stimuli (Rottenberg et al., 2002, 2005). In addition, trait and state NA appear to be associated with difficulties disengaging attention from salient, distracting information (Compton, 2000; Bredemeier et al., 2011). The present study therefore included both positive and negative stimuli matched on arousal.

## Materials and Methods

### Participants

Participants were recruited from the local community via advertisements, gave written informed consent, and participated in the study, which was approved by the Institutional Review Board of the University of Illinois at Urbana-Champaign. All participants were right-handed, native speakers of English with self-reported normal color vision and no reported neurological disorders or impairments. Participants were excluded if they exhibited current substance abuse or dependence, mania, or psychosis, as assessed by the Structured Clinical Interview for the DSM-IV. Participants were given a laboratory tour, informed of the procedures of the study, and screened for claustrophobia and other contraindications for MRI participation. Thirty participants were excluded from analyses for a variety of reasons, including excessive motion in the scanner, technical errors during fMRI acquisition, loss of questionnaire or RT data, outliers on the questionnaires or in RT (outliers were defined as greater than 3 standard deviations from the mean), or error rates exceeding 15%. The final sample included 101 paid participants (62 females, age *M *= 34.57, SD = 9.27).

### Questionnaires

During the laboratory tour, participants completed the 28-item Negative Temperament scale of the General Temperament Survey (GTS-NT) to assess trait NA (Watson and Clark, 1993). Participants were instructed to decide whether each statement mostly described them and to rate each item as true or false. Sample items include “I often have strong feelings such as anxiety or anger without really knowing why,” “I sometimes get all worked up as I think about things that happened during the day,” and “Often life feels like a big struggle.” State NA was measured using the NA scale from the Positive and NA Schedule (PANAS-NA; Watson et al., 1988), which was administered immediately before participants performed the emotion-word Stroop task during fMRI. Participants indicated the extent to which they were feeling each of 10 negative emotions (e.g., afraid, nervous, irritable, upset) that day on a scale from 1 (“*very slightly or not at all*”) to 5 (“*extremely*”).

Participants also completed measures of anxiety and depression during the laboratory tour. These measures were used in analyses to determine whether results depended on anxiety and depression. The 16-item Penn State Worry Questionnaire (PSWQ) was used to assess anxious apprehension or worry (Meyer et al., 1990; Molina and Borkovec, 1994). Participants responded to questions such as “Many situations make me worry,” by rating how characteristic each statement was of them on a scale from 1 (“*not at all typical*”) to 5 (“*very typical*”). Participants also completed the Anxious Arousal and Anhedonic Depression subscales of the Mood and Anxiety Symptom Questionnaire (MASQ), rating how much they experienced each item during the previous week on a scale from 1 (“*not at all*”) to 5 (“*extremely*”; Watson et al., 1995a,b). The MASQ Anxious Arousal subscale (MASQAA) consists of 17 items in which participants responded to statements such as “Heart was racing or pounding.” The eight-item MASQ Anhedonic Depression subscale (MASQAD8) was used as it has been shown to reflect depressed mood (Nitschke et al., 2001) and to predict current and lifetime depressive disorders (Bredemeier et al., 2010). The MASQAD8 scale consists of items such as “Felt like nothing was very enjoyable.”

### Stimuli and experimental design

Participants performed two tasks, a color-word Stroop and an emotion-word Stroop, during the fMRI session and also in a similar EEG session. Only fMRI data from the emotion-word Stroop task are reported here. The order of the Stroop tasks within session and the order of fMRI and EEG sessions were counterbalanced. The emotion-word Stroop task employed a block design consisting of blocks of positive or negative emotion words alternating with blocks of neutral words. Each participant received one of eight orders designed to ensure that the blocks of emotional and neutral words preceded each other equally often. There was a brief rest period after every fourth block. Additionally, there were four fixation blocks (one at the beginning, one at the end, and two in the middle) in which a brighter fixation cross was presented for 1500 ms, followed by a dimmer fixation cross for an average of 500 ms.

Participants received 256 trials in 16 blocks (four positive, eight neutral, four negative) of 16 trials, with a variable ITI averaging 2000 ms (±225 ms) between trial onsets. A trial began with the presentation of a word for 1500 ms, followed by a fixation cross for an average of 500 ms. Each trial consisted of one word presented in one of four ink colors (red, yellow, green, blue). Participants were instructed to press one of four buttons to indicate the color of the ink in which the word appeared on the screen, while ignoring the word meaning (thus making word meaning irrelevant to the task). Each color occurred equally often with each word type (positive, neutral, negative), and trials were pseudorandomized such that no more than two trials featuring the same color appeared in a row. Participants completed 32 practice trials before starting the main task.

The 256 emotion-word stimuli were selected from the Affective Norms for English Words (ANEW) set (Bradley and Lang, 1999). Sixty-four positive (e.g., birthday, laughter), 64 negative (e.g., suicide, war), and two sets of 64 neutral (e.g., hydrant, moment) words were carefully selected on the basis of established norms for valence, arousal, word length, and frequency of use in the English language (Bradley and Lang, 1999). Both positive and negative words were selected to be highly arousing, whereas the neutral words were selected to be low in arousal (see Herrington et al., 2010, for detailed stimulus characteristics). Stimuli were displayed using back projection, and word presentation and reaction time (RT) measurement were controlled by STIM software (James Long Company, Caroga Lake, NY, USA).

### Image acquisition

MR data were collected using a 3-T Siemens Allegra scanner. Gradient field maps were collected to correct for geometric distortions in the functional data caused by magnetic field inhomogeneity (Jezzard and Balaban, 1995). Three hundred and seventy functional images were acquired using a Siemens gradient-echo echo-planar imaging sequence (TR 2000 ms, TE 25 ms, flip angle 80°, FOV 22 cm). Thirty-eight oblique axial slices (slick thickness 3 mm, in-plane resolution 3.4375 mm × 3.4375 mm, 0.3 mm gap between slices) were acquired parallel to the anterior and posterior commissures. After the functional acquisition, an MPRAGE structural sequence was also acquired (160 axial slices, slice thickness 1 mm, in-plane resolution 1 mm × 1 mm) for registering each participant’s functional data to standard space.

### fMRI data reduction and analyses

Image processing and statistical analyses were implemented primarily using the FSL analysis package^1^. Functional data for each participant were motion-corrected using rigid-body registration via FMRIB’s linear registration tool MCFLIRT (Jenkinson et al., 2002). Spikes or sudden intensity shifts were corrected using AFNI’s 3dDespike program^2^. The time series of one participant was truncated due to excessive motion only at the end of the scan. All other participants demonstrated less than 3.3 mm absolute motion or 2 mm relative motion (participants with motion exceeding this threshold were excluded from analysis, leaving *N* = 101). After motion correction and despiking, each time series was corrected for geometric distortions caused by magnetic field inhomogeneity. Remaining preprocessing steps, single-subject statistics, and higher-level regression analyses were done with FEAT (FMRI Expert Analysis Tool, FMRIB’s Software Library^3^). The first three fMRI volumes of each time series were discarded in order to allow the MR signal to reach a steady state. The data were then intensity-normalized, temporally filtered with a high-pass filter, and spatially smoothed using a 3D Gaussian kernel (FWHM = 5 mm).

Regression analyses were then performed on each participant’s time series using FILM, FMRIB’s Improved Linear Model with autocorrelation correction (Woolrich et al., 2001). Statistical maps were generated via multiple regression computed for each intracerebral voxel. Four explanatory variables were created for each condition (positive, neutral, negative, and rest) and included in the regression model, with fixation left as the unmodeled baseline. Each explanatory variable was convolved with a gamma function to approximate the temporal course of the blood-oxygen-level-dependent (BOLD) hemodynamic response function. Each explanatory variable yielded a per-voxel effect-size parameter estimate (β) map representing the magnitude of activation associated with that explanatory variable. In order to create comparisons of interest, β values for the relevant parameters were contrasted. As is customary in our laboratory research (for further discussion, see Herrington et al., 2010), data were analyzed with respect to positive affect (positive vs. neutral words), NA (negative vs. neutral words), arousal (positive and negative words vs. neutral words), and valence (negative vs. positive words). Because preliminary analyses indicated that the effects of interest were similar for both positive and negative words, this report will focus on the arousal contrast. However, the orthogonal valence contrast (negative vs. positive words) is also reported to indicate that the effects in brain regions of interest (e.g., posterior vs. mid-DLPFC) were similar for both positive and negative stimuli.

For each participant, the functional activation maps were warped into a common stereotaxic space [the 2009 Montreal Neurological Institute (MNI) 152 symmetrical 1 mm × 1 mm × 1 mm template; Fonov et al., 2009] using FMRIB’s Non-Linear Image Registration Tool, FNIRT (Andersson et al., 2007). First, the middle volume of the functional scan was registered to the structural scan using rigid-body registration (only allowing *xyz* translation and rotation). Next, the structural scan was registered to the MNI template using a two-step process. First, a linear registration was carried out, allowing xyz translation, rotation, zoom, and shear. Finally, a non-linear registration using cubic b-spline basis functions was carried out, with the results of the linear registration as a starting point. The three registration steps (rigid-body function to structural, affine structural to MNI, and non-linear structural to MNI) were concatenated to create a warp that mapped functional to MNI space and was then applied to the β maps.

Cross-subject inferential statistical analyses of brain activation were carried out using FLAME (FMRIB’s Local Analysis of Mixed Effects). The arousal contrast was entered as a dependent variable (DV) in a multiple regression analysis with questionnaire scores (GTS-NT scale for trait NA and PANAS-NA scale for state NA) entered as continuous predictors to predict activation voxel-by-voxel. Two different higher-level analysis approaches served to identify (1) brain areas associated with both trait and state NA and (2) brain areas showing distinct relationships with them. First, separate regressions were performed for each questionnaire (without the shared variance from the other questionnaire removed). These essentially provided zero-order correlations between trait or state NA and activation in each brain voxel. These were followed by a conjunction analysis to reveal areas in common for trait and state NA (Nichols et al., 2005). A conjunction *z* map was created by comparing the *z* value of each voxel in the trait NA map with the *z* value in the state NA map. If the *z* scores were of opposite signs, the value for the voxel in the conjunction *z* map was set to zero. If the *z* scores were in the same direction, the value for the voxel in the conjunction *z* map was assigned to the *z* value with the weaker significance. The conjunction *z* map was then thresholded to identify significant clusters, using the thresholding method described below. Second, the GTS-NT and PANAS-NA scores were entered simultaneously as predictors into a higher-level regression analysis. The resulting β map for each predictor reflected the unique variance associated with that predictor. Because the conjunction analyses identified no shared brain regions, and results for the zero-order correlations in the first set of analyses generally resembled results from the second set of analyses, the latter are reported below. The interaction between trait and state NA was added as a third independent variable (IV) to this latter analysis to examine regions where the relationship between trait NA and brain activation depended on the level of state NA.

Significantly activated voxels were identified via thresholding of per-voxel *t*-tests conducted on contrast βs maps that were converted to *z* scores. All hypotheses regarding the main effects of trait and state NA were directional, justifying one-tailed tests for these analyses. Monte Carlo simulations via AFNI’s AlphaSim program were used to estimate the overall significance level (probability of a false detection) for thresholding the 3D functional *z* map image (Ward, 2000). The simulations provided the appropriate cluster size to give an overall family wise error rate of *p* ≤ 0.05 (although all clusters reported here survived a more stringent family wise error rate of *p* = 0.01).

To limit the number of voxels under consideration, *a priori* regions of interest were examined using masks of the frontal cortex, ACC, amygdala, and parietal cortex that were created using the Harvard-Oxford probabilistic atlas available with FSL. For each of these masks, a cluster size threshold was computed and used only for voxels within the mask. An individual voxel level threshold *z* value of 2.0537 was used for all masks. The minimum cluster sizes for the masks were: frontal cortex = 702 mm^3^, ACC = 351 mm^3^, amygdala = 234 mm^3^, and parietal cortex = 780 mm^3^. A whole-brain gray-matter mask was used to examine areas not involved in *a priori* hypotheses (cluster size threshold = 1170 mm^3^). All analyses were also conducted using two-tailed tests, with results largely in line with the planned one-tailed tests. In no case did a two-tailed test result in significant clusters in the direction opposite to hypotheses. Two-tailed results for these analyses are thus not reported here. Because the analysis examining the interaction between trait and state NA was exploratory, two-tailed tests using the whole-brain gray-matter mask were conducted (cluster size threshold = 2340 mm^3^).

### Lateralization analyses

Research supports important distinctions in functional specialization of the two hemispheres, particularly the frontal cortex (e.g., Herrington et al., 2005, 2010; Engels et al., 2007, 2010; Spielberg et al., 2011; for reviews, see Heller et al., 1998; Herrington et al., 2009). Therefore, analyses were conducted for the clusters that emerged in the frontal cortex to determine whether they were significantly lateralized. Lateralization was tested using a locally written Matlab program (Spielberg et al., 2011). This program implemented a repeated measures homogeneity of slopes General Linear Model, with hemisphere as the repeated measure, trait and state NA scores as continuous predictors, and fMRI activation for the arousal contrast as the DV. This ANCOVA was conducted on a per-voxel basis, and the resultant β maps were thresholded as described above, with the exception that *F*-tests were used. Because testing laterality determines whether the β in a voxel in the right hemisphere differs significantly from the β in the homologous voxel in the left hemisphere, half as many tests are conducted as in a full-brain analysis. Therefore, a mask containing only the right frontal hemisphere was created.

### Behavioral data

Average RT and number of errors were computed for each condition (positive, neutral, and negative). An ANOVA examined RT differences across conditions, and a Friedman test determined whether the number of errors differed across conditions. An arousal interference score was calculated by subtracting each participant’s average neutral-word RT from the mean of their positive-word and negative-word RT averages. Higher interference scores indicate that participants took longer to respond to emotionally arousing words than neutral words. To examine the relationship between arousal interference and trait and state NA, arousal RT interference was entered as a DV in regression analyses first with each questionnaire entered separately and second with the questionnaires entered simultaneously as predictors. The interaction between trait and state NA then was added to this latter analysis to examine whether the relationship between trait NA and arousal interference depended on the level of state NA. Although RT interference was the primary behavioral measure of interest, Poisson regression analyses were also conducted using number of task errors as the DV.

### Analysis of brain activation and behavior relationships

In order to explore the relationship between behavioral performance and neural activation in the clusters associated with trait and state NA, β values were averaged across all voxels in each cluster to create a single score for each cluster for each participant. Correlations between arousal RT interference and cluster scores, as well as number of task errors and cluster scores, were calculated using PASW Statistics (SPSS) 18.

### Mediation analyses

A multiple mediation analysis was conducted *post hoc* to follow-up results from primary analyses in order to better understand relationships between brain activation and behavior. The multiple mediation model involves an IV, a DV, and *j* possible mediators (M). In the present model, the IV was left posterior DLPFC, the DV was arousal RT interference, and the possible mediators were the rostral anterior cingulate cortex (rACC), precuneus, and caudate clusters that emerged from the trait NA analysis (see below). The multiple mediation model allows for the testing of several potential mediators simultaneously and has several advantages over testing separate simple mediation models. It can be used to determine (1) whether an overall mediation effect exists (analogous to conducting a regression analysis with multiple predictors and evaluating total *R*^2^) and (2) whether specific variables uniquely mediate the direct effect (conditional on other mediators being included in the model). It also reduces the likelihood of parameter bias due to omitted variables (see Preacher and Hayes, 2008, for discussion).

Following the recommendation of Preacher and Hayes (2008), mediation (examined via significance of the indirect effect a*_j_* × b*_j_*) was determined by using bootstrapped confidence intervals rather than the Sobel (1982) test. The bootstrap method is preferred over the Sobel test because the former does not require the assumption of a normal distribution, and simulations have shown that bootstrapping methods have higher power while still performing well regarding Type I error rates (MacKinnon et al., 2002, 2004). The SPSS macro script of Preacher and Hayes (2008) was used to conduct multiple mediation analyses by calculating 95% bias-corrected and accelerated bootstrap confidence intervals for the indirect effect involving 5,000 repetitions.

## Results

### Behavioral performance

A repeated measures ANOVA [*F*(2, 98) = 12.38, *p* < 0.001, with Huynh–Feldt correction] and *post hoc* paired *t*-tests indicated that RT for negative words (*M* = 721 ms, SD = 100 ms) was longer than RT for both neutral (*M* = 704 ms, SD = 99 ms) and positive words (*M* = 703 ms, SD = 96 ms). A Friedman non-parametric test [χ^2^(2) = 10.50, *p* < 0.01] and *post hoc* analyses with Wilcoxon Signed Ranks Tests showed that individuals made more errors in the neutral-word condition (Md = 4, 2–6) than both the positive-word (Md = 3, 2–4) and negative-word conditions (Md = 3, 2–5).

Table 1 lists the results of the regression analyses for the behavioral data. State NA was associated with arousal-related RT interference and total number of task errors. Higher levels of state NA were associated with (1) responding more slowly to emotionally arousing words than to neutral words and (2) committing more errors. Trait NA was not associated with arousal RT interference or errors. The interaction between trait and state NA predicted task errors, such that increased trait NA was associated with increased task errors at high levels of state NA, but with decreased errors at low levels of state NA.

**Table 1:** **Regression analyses for behavioral data**.

Variable	DV = Arousal RT interference		
	Beta^t^	*p*	*R*^2^
**ENTERED SEPARATELY**			
GTS NT (trait NA)	−0.04	0.70	0.00
PANAS-NA (state NA)	0.20	0.04	0.04
**ENTERED SIMULTANEOUSLY**			
Step 1
GTS NT	−0.10	0.32	0.05
PANAS-NA	0.23	0.03	
Step 2
GTS NT × PANAS-NA	−0.28	0.62	0.05

**Variable**	**DV = Total task errors**		
	**Beta**	**Wald χ^2^**	***p***

**ENTERED SEPARATELY**			
GTS NT	0.01	4.26	0.04
PANAS-NA	0.04	16.08	0.00
**ENTERED SIMULTANEOUSLY**			
Step 1
GTS NT	0.01	1.04	0.31
PANAS-NA	0.04	12.66	0.00
Step 2
GTS NT × PANAS-NA	0.01	11.67	0.00

*^t^Standardized betas for RT interference analyses; GTS NT, general temperament survey negative temperament scale; PANAS-NA, positive and negative affect schedule negative affect scale*.

### Brain regions uniquely associated with trait negative affect

**Figure 1:**
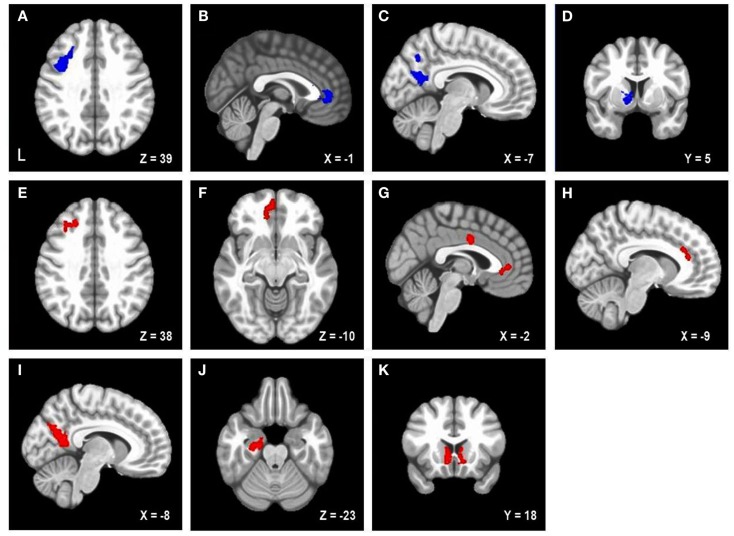
**Areas uniquely associated with either trait or state negative affect (NA)**. Blue = decreased brain activation associated with trait NA **(A–D)**. Red = increased brain activation associated with state NA **(E–K)**. L, left. Clusters of activation in **(A)** Left posterior DLPFC, **(B)** Rostral anterior cingulate cortex (rACC), **(C)** Precuneus, **(D)** Left caudate, **(E)** Left mid-DLPFC, **(F)** Left medial frontal cortex, **(G)** rACC and posterior dorsal ACC (dACC), **(H)** dACC, **(I)** Precuneus, **(J)** Parahippocampal gyrus, **(K)** Bilateral nucleus accumbens/caudate.

Table 2 lists the four brain regions that were negatively correlated with trait NA. In line with hypotheses, higher levels of trait NA were associated with less brain activation in left posterior DFPFC [middle frontal gyrus (MFG) extending into precentral gyrus], rACC, and precuneus (see Figure 1). In addition, a cluster emerged in left caudate when using the whole-brain gray-matter mask. There were no significant amygdala clusters. Further, there were no significant clusters positively correlated with trait NA. An examination of the valence contrast (negative vs. positive words) confirmed that there were no significant activations overlapping with any of these four areas, indicating that these results were not driven by negative or positive words alone.

**Table 2:** **Brain areas moderated by trait and state negative affect and correlations with behavior**.

Region	Cluster size (mm^3^)	Mean *z* value	Location			*r*_RT_
			*X*	*Y*	*Z*	
**TRAIT NEGATIVE AFFECT**						
L middle frontal gyrus/precentral gyrus (posterior DLPFC)^a^	3,200	−2.35	−34	16	39	0.38**
Rostral anterior cingulate cortex^b^	1,430	−2.41	−3	40	0	0.27**
Precuneus^c^	3,902	−2.36	−3	−60	22	0.45**
L caudate^d^	2,678	−2.29	−10	5	3	0.43**
**STATE NEGATIVE AFFECT**						
L middle frontal gyrus/inferior frontal gyrus (mid-DLPFC)^a^	1,182	2.35	−26	29	40	0.37**
L medial frontal cortex^a^	1,124	2.31	−6	52	−10	0.42**
Rostral anterior cingulate cortex^b^	547	2.28	0	39	2	0.26**
Dorsal anterior cingulate cortex^b^	390	2.30	−9	33	21	0.36**
Posterior dorsal anterior cingulate cortex^b^	586	2.33	−2	−3	36	0.26**
Precuneus^c^	5,471	2.48	−4	−62	21	0.46**
L parahippocampal gyrus^d^	1,636	2.42	−21	−14	−25	0.24*
L & R nucleus accumbens/caudate^d^	3,230	2.45	−1	14	−2	0.46**

L, left; R, right; Location, Coordinates are for the center-of-mass in MNI152 2009a symmetrical space; r_RT_, correlation between reaction time interference and activation in each ROI; ^a^Correction for only frontal cortex voxels; ^b^Correction for only anterior cingulate cortex voxels; ^c^Correction for only parietal cortex voxels; ^d^Correction for all gray-matter voxels. *p < 0.05; **p < 0.01.

Follow-up analyses were conducted to determine whether these effects depended on anxiety and depression. PSWQ, MASQAA, and MASQAD8 scores were included in a multiple regression analysis along with trait and state NA scores (*N* = 99 due to missing PSWQ data for two individuals). All of the results remained significant for trait NA, with the exception of the caudate.

### Brain regions uniquely associated with state negative affect

Table 2 lists the regions that were positively correlated with state NA. In line with hypotheses, higher levels of state NA were associated with more activation in mid-DLPFC [MFG, extending into inferior frontal gyrus (IFG)], ACC, and precuneus (see Figure 1). Three separate clusters emerged in the ACC: rACC, an anterior region of dorsal ACC (dACC), and posterior dACC. In addition to mid-DLPFC, a second cluster emerged using the frontal cortex mask, in left medial frontal cortex. Finally, when using the whole-brain gray-matter mask, two additional clusters emerged: one in left parahippocampal gyrus and one spanning left and right nucleus accumbens/caudate. There were no significant amygdala clusters. Further, there were no significant clusters negatively correlated with state NA. An examination of the valence contrast (negative vs. positive words) confirmed that there were no significant activations overlapping with any of these eight areas.

Follow-up analyses were conducted to determine whether these effects depended on anxiety and depression. All of the results remained significant for state NA when PSWQ, MASQAA, and MASQAD8 were added to the regression analysis.

### The interactive effects of trait and state negative affect

**Figure 2:**
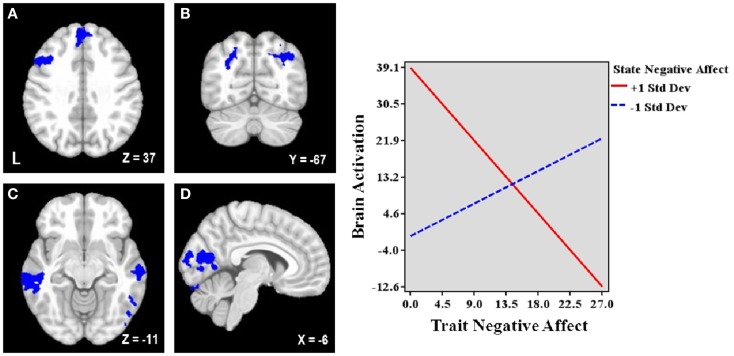
**Brain areas associated with the interaction between trait and state negative affect (NA)**. Blue = Less activation when both dimensions are high than when one dimension is high. L, left. Clusters of activation in **(A)** Left lateral middle frontal gyrus (MFG) and medial superior frontal gyrus, **(B)** Bilateral parietal cortex/occipital cortex, **(C)** Bilateral middle temporal gyrus, **(D)** Occipital cortex. Graphing the two-way interaction for each region shows that trait NA’s relationship with these brain areas depends on the level of co-occurring state NA, such that increased trait NA is associated with decreased activation in all of these areas at high levels of state NA but with increased activation at low levels of state NA. Depicted is a representative graph from left MFG.

Table 3 lists the seven regions negatively associated with the interaction between trait and state NA. These regions include left DLPFC (lateral MFG), medial superior frontal gyrus (SFG), bilateral superior parietal cortex (extending into occipital cortex), bilateral middle temporal gyrus (MTG), and occipital cortex (spanning intracalcarine cortex/lingual gyrus/occipital fusiform gyrus; see Figure 2). Graphing the interactions for all brain areas showed that increased trait NA was associated with decreased activation in these areas at high levels of state NA but with increased activation at low levels of state NA (see Figure 2). No regions were positively correlated with the interaction between trait and state NA. An examination of the valence contrast (negative vs. positive words) indicated that there were no significant activations overlapping with any of these areas.

**Table 3:** **Brain areas with interactive effects for trait and state negative affect and correlations with behavior**.

Region	Cluster size (mm^3^)	Mean *z* value	Location			*r*_RT_
			*X*	*Y*	*Z*	
L lateral middle frontal gyrus^a^	2,828	−2.52	−44	16	37	0.32**
Medial superior frontal gyrus^a^	2,750	−2.37	−1	44	38	0.37**
L superior parietal cortex/occipital cortex^a^	2,594	−2.42	−26	−72	39	0.12*
R superior parietal cortex/occipital cortex^a^	2,424	−2.38	33	−69	45	0.14
L middle temporal gyrus^a^	7,422	−2.60	−61	−37	−4	0.43**
R middle temporal gyrus^a^	6,990	−2.43	58	−46	−3	0.22*
Occipital cortex (intracalcarine cortex/lingual gyrus/occipital fusiform gyrus)^a^	24,198	−2.43	−4	−74	−5	−0.04

L, left; R, right; Location, Coordinates are for the center-of-mass in MNI152 2009a symmetrical space; *r*_RT_, correlation between reaction time interference and activation in each ROI; ^a^, Correction for all gray-matter voxels 2-tailed test. *p < 0.05; **p < 0.01.

### Lateralization analyses

Lateralization analyses were conducted to explore whether the three left frontal clusters (posterior DLPFC associated with trait NA, mid-DLPFC/IFG associated with state NA, and lateral MFG associated with their interaction) were significantly lateralized. Only the lateral MFG cluster associated with the interaction between trait and state NA was significantly left-lateralized.

### Correlations between brain activation and behavioral performance

As presented in Table 2, all clusters associated with trait and state NA were positively correlated with RT to high-arousing vs. neutral stimuli. These positive correlations indicate that, as activation in these areas increased, participants were more distracted by the emotional nature of the words. Furthermore, as seen in Table 3, five of the seven clusters associated with the interaction between trait and state NA were also positively correlated with RT interference from arousing words, including left DLPFC (MFG), medial SFG, left superior parietal cortex, and left and right MTG. Only the cluster located in mid-DLPFC (positively associated with state NA) was marginally correlated with error rate (*r* = 0.17, *p* = 0.08). As activation in this area increased, participants made more errors.

### Mediation analyses

**Figure 3:**
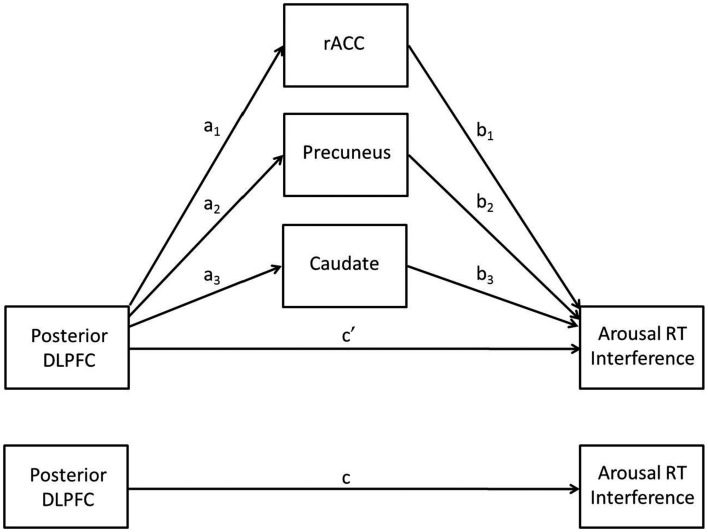
**The multiple mediation model**.

Less activation in posterior DLPFC for higher levels of trait NA was consistent with hypotheses. It was also expected that less activation in this region would be associated with decrements in performance, such that individuals with high levels of trait NA would have more difficulty ignoring the arousing nature of the stimuli given their difficulty recruiting posterior DLPFC to implement top-down attentional control. However, the positive correlation between brain activation in this area and RT interference indicated that less activation in posterior DLPFC was associated with better performance (less distraction by the emotional words). A multiple mediation analysis (see Figure 3) was conducted to investigate the possibility that the impact of posterior DLPFC activation on behavioral performance was actually mediated by other brain areas. This hypothesis is in line with the cascade-of-control model, which posits that the ACC comes online after the DLPFC in order to implement response-related attentional processes, such as evaluating responses.

The results of the mediation analysis are presented in Table 4. The total indirect effect was significant, as was each of the specific indirect effects, indicating that the rACC, precuneus, and caudate clusters each mediated the relationship between posterior DLPFC activation and RT interference. As shown in the table, the effect of posterior DLPFC on RT interference was reduced from 0.67 to 0.06 by the three mediators, going from a significant (*p* = 0.0001) to a non-significant (*p* = 0.84) relationship, indicating full mediation. Consistent with the cascade-of-control model, rACC mediated the relationship between DLPFC and RT interference. Importantly, the effect of rACC on RT interference (path b) was negative, indicating that less activation in rACC was associated with more interference from arousing stimuli (when partialing out the effect of the posterior DLPFC). Thus, less activation in posterior DLPFC is associated with less activation in rACC, which is in turn associated with difficulty ignoring emotionally arousing stimuli.

**Table 4:** **Summary of multiple mediation analysis**.

IV = L posterior DLPFC	DV = Arousal RT interference				
Mediators (M)	Path a (IV to M)	Path b (M to DV)^a^	Path c (IV to DV total)	Path c′ (IV to DV direct)	a × b (Indirect effect)
rACC	1.29**	−0.29*			−0.37*
Precuneus	1.06**	0.51**			0.54**
Caudate	1.34**	0.33*			0.45*
			0.67**	0.06	
					Total indirect effect: 0.61*

^a^Partialing out effects of IV; *p ≤ 0.05; **p < 0.01.

## Discussion

Overall, results of the present study indicate that trait NA, state NA, and their interaction have unique correlates in a selective attention task involving emotional distraction. The pattern of brain activation associated with trait NA suggests that individuals high in trait NA have difficulty engaging top-down aspects of attentional control to maintain task goals in the presence of irrelevant information (Banich, 2009; Herrington et al., 2010). As hypothesized, trait NA was associated with less activation to emotionally arousing stimuli in posterior DLPFC, which plays a key role in implementing top-down attentional control in order to ignore distracting information and focus on the task at hand (Banich et al., 2000a,b, 2009; Compton et al., 2003; Milham et al., 2003). This finding is consistent with studies that found less activity in this area in individuals high in anhedonic depression in the context of negative vs. neutral words (Engels et al., 2010; Herrington et al., 2010). The present finding suggests that abnormal activation in this area is not specific to depression but is associated with trait NA, a more general risk factor for developing and maintaining anxiety and depression (Ormel et al., 2004). Further, individuals high in trait NA had difficulty ignoring salient, arousing information, both positively and negatively valenced, suggesting that their attentional deficit was not limited to negative stimuli. Consistent with this finding, neuroticism has been associated with difficulty ignoring salient distracters during a non-emotional task (Bredemeier et al., 2011).

Corbetta et al. (2008) proposed that posterior portions of the frontal cortex are part of a dorsal frontoparietal network involved in the top-down selection of stimuli congruent with goals and expectations based on previous experiences. Further, posterior DLPFC feeds signals to a separate ventral attentional network that detects salient stimuli in order to bias processing of the appropriate stimulus features (Corbetta et al., 2008). Given that individuals high in trait NA are theorized to rely excessively on pre-existing knowledge and ignore meaningful contextual details (Robinson and Clore, 2002), dysfunction in a key node of the dorsal attentional network may help explain their difficulty integrating pre-existing information with immediate environmental information most relevant for current goals. Functional impairments in posterior DLPFC also perturb activity in parietal regions that it modulates. In the present study, less activation in a parietal region associated with trait NA, the precuneus, suggests that this area failed to receive signals from posterior DLPFC to bias processing toward task-relevant aspects of stimulus representations (color information) and away from irrelevant features (word meaning; Banich et al., 2000b). Further, the precuneus is a central hub linking the frontal cortex with other parietal regions (Bullmore and Sporns, 2009). Thus, dysfunction in the precuneus may prevent successful modulation of other key parietal areas.

The cascade-of-control model (Banich, 2009) asserts that attentionally demanding tasks first recruit DLPFC, which in turn influences later ACC activity. Silton et al. (2010) confirmed this hypothesized within-trial sequence using fMRI-guided event-related potential (ERP) source localization. In the present study, high trait NA was associated with hypoactivity in rACC, a region involved in evaluating interference from emotionally salient distracters (Whalen et al., 1998; Vuilleumier et al., 2001; Mohanty et al., 2007). The combination of hypoactivity in both posterior DLPFC and rACC regions in individuals high in trait NA suggests a mechanism by which they have difficulty exerting top-down control to handle conflict from emotional distracters. Further, the finding that rACC activation mediated the relationship between posterior DLPFC activation and RT interference suggests that DLPFC failed to recruit rACC to compensate for poor attentional control, thus leading to increased distraction from emotionally arousing stimuli. Similar deficits in the fronto-cingulate network have been observed in anxious individuals (Bishop et al., 2004; Engels et al., 2007) and may be related to high levels of trait NA rather than anxiety-specific symptoms.

In contrast to trait NA, the pattern of brain activity associated with state NA suggests that, when individuals are in negative moods, they engage in excessive processing of salient stimuli. This is problematic when their performance is penalized due to constant interruption of top-down processing to focus on stimuli features that are not task-relevant. State NA was associated with increased activation in mid-DLPFC, an area involved in stimulus-driven attentional control. Previous studies support the role of mid-DLPFC in selecting and processing the most pertinent aspects of stimuli in the environment and ignoring irrelevant features (for review, see Herrington et al., 2005, 2010; Engels et al., 2007; Banich, 2009). Further, this anterior portion of MFG has been proposed to be part of a stimulus-driven ventral attentional network (Corbetta et al., 2008). In the present study, the cluster in mid-DLPFC extended ventrally into IFG, another key node in this network. This network detects salient stimuli in the surroundings and determines their behavioral relevance, as well as interrupts top-down processing in order to reorient attention to stimuli that have been determined to be “important” (Corbetta et al., 2008). Hyperactivity in mid-DLPFC may indicate that individuals high in state NA were over-attending to the emotional aspects of the words, which impeded their ability to focus on the ink color (Engels et al., 2010). This explanation is supported by the significant relationship between increased activity in mid-DLPFC and increased behavioral (RT) interference for emotionally arousing than for neutral words. Mid-DLPFC activation was also marginally positively correlated with overall task errors.

State NA was also associated with three clusters in ACC: one in anterior dACC, one in posterior dACC, and one in rACC. The anterior portion of dACC appears to be involved in response evaluation (Milham and Banich, 2005; Banich, 2009), whereas the posterior portion plays a critical role in response selection (Milham et al., 2001, 2003; Banich, 2009). Further, if DLPFC function earlier in the processing stream is problematic, posterior dACC must deal with unresolved selection issues (e.g., Milham et al., 2002; Silton et al., 2010). In the present study, increased activation in both portions of dACC was associated with increased interference from emotional stimuli. Increased dACC activity may reflect (1) unsuccessful attempts to compensate for dysfunctional DLPFC control and (2) attempts to signal the DLPFC to engage stronger top-down control in the future, in order to override stimulus-driven processing of irrelevant information (Milham et al., 2003; Banich, 2009). A third cluster located in rACC overlapped with the cluster that emerged in the trait NA analysis. However, the relationship between state NA and rACC was in the opposite direction of trait NA and rACC, such that increased levels of state NA were associated with increased activation in rACC. In addition to being involved in attentional control during emotional tasks, rACC has been implicated in regulating responses to emotional material (Bush et al., 2000).

A cluster in medial frontal cortex also emerged in the state NA analysis. This region has been implicated in various functions related to affective states, including responding to emotional pictures and making attributions about emotional states (Lane and McRae, 2004; Ochsner et al., 2004, 2009). Increased activation in both rACC and medial frontal cortex in the present study for individuals high in state NA suggests that they were involved in attending to the emotional content of the stimuli and/or their own mood states. State NA was also associated with activation in precuneus and parahippocampal gyrus. Given that precuneus activation was associated with difficulty ignoring the emotional content of the words, it is likely that individuals high in state NA were biased toward the processing of word meaning instead of word color. Previous studies of the Stroop task found decreased parahippocampal activity was associated with successful implementation of attentional control and proposed that it reflected inhibited binding of ink color and word meaning (Compton et al., 2003). Increased activation in the hippocampal gyrus in the present study may indicate that individuals high in state NA were not inhibiting word meaning optimally, which contributed to detriments in their performance.

The results of the present study indicate that co-occurring high levels of trait and state NA are associated with a distinct pattern of brain activation, beyond the additive effects of trait and state NA. The clusters that emerged for the interaction analysis of trait and state NA did not overlap with any of the clusters associated with the main effects, highlighting the importance of considering the interactive effects of these constructs. Relative to being high in only one dimension, being high or low in both trait and state NA was associated with less activation in lateral MFG and medial SFG, indicating difficulty maintaining a top-down, goal-congruent task set while dealing with distracting emotional information (Ferstl et al., 2005; Spielberg et al., 2011). The combination of high trait and state NA was also associated with decreased activation in several areas early in the dorsal processing stream, including occipital cortex, bilateral MTG, and bilateral superior parietal areas. Hypoactivation in frontal areas as well as in visual and temporal areas suggests that these latter regions were not appropriately modulated by the prefrontal cortex to bias processing of the relevant sensory representations (Banich et al., 2000b). Decreased activation in superior parietal cortex suggests that combined trait and state NA will lead to more difficulty allocating attentional resources in a top-down manner than high trait or state NA alone (Husain and Nachev, 2007; Corbetta et al., 2008).

In summary, state and trait NA appear to disrupt attentional control in distinct ways. Individuals with high trait NA appear to have difficulty sustaining attention and persisting in goal achievement in environments involving salient distractions, as well as trouble anticipating and preparing for upcoming tasks (Corbetta et al., 2008). Present results are consistent with research indicating that high trait anxious individuals exhibit problems engaging proactive control, or “sustaining representation of task requirements or goals throughout periods of high control demand” (Fales et al., 2008, p. 240), though such deficits may not be specific to anxiety. Difficulty implementing top-down attentional control in the face of distracting emotional information likely contributes to the biased expectations, interpretations, and attributions of environmental information associated with high levels of trait NA. Individuals high in trait NA appear to rely excessively on (potentially inappropriate) knowledge based on previous experiences, failing to integrate it with pertinent information in the current context in order to respond adaptively.

In contrast, high levels of state NA are associated with an over-reliance on more transient aspects of attentional control and excessive processing of salient information. Hyperactivity of a key node in a stimulus-driven attentional network likely leads to repeated interruption of top-down processing to focus on task-irrelevant contextual details. Whereas individuals with high trait NA appear to be ineffective at incorporating information from their immediate environment, those high in state NA appear to have the opposite problem, such that they have difficulty appropriately attending to ongoing goals. The present findings support the assertion that trait NA is not simply the tendency to experience negative states; rather, it has correlates dissociable from state NA. Further, present results contribute to the understanding of the psychological and biological mechanisms through which trait and state NA trigger and maintain symptoms of anxiety and depression.

The present study benefited from a sample size that is unusually large for the fMRI literature, as well as an analysis strategy that allowed for the examination of the distinct correlates of trait and state NA, rather than confounding their effects. Given that trait and state NA are moderately correlated, measuring just one dimension may reflect effects that are not specific to that dimension and can be misleading. Further, the study extends the literature by examining attentional control in the context of distracting emotional stimuli that are both positively and negatively valenced, equated for arousal levels.

Limitations include a correlational design, which cannot determine whether trait and state NA lead to the development of attentional control difficulties or vice versa. In addition, due to the nature of the task and methods used, distinct stages of attention (e.g., orientating vs. disengaging) could not be examined to determine whether trait and state NA are associated with difficulties at different stages. Future research could employ other attentional tasks and utilize ERP methods to address this question. Another empirical question that remains to be tested is the extent to which these results generalize to other types of emotional stimuli (e.g., faces, emotional scenes), as it is likely that there would be some differences (e.g., Isaac et al., 2012). Finally, future work should also examine tasks that involve salient, non-emotional distracters in order to determine whether these attentional control deficits are specific to emotional information.

## Conflict of Interest Statement

The authors declare that the research was conducted in the absence of any commercial or financial relationships that could be construed as a potential conflict of interest.

## Footnotes

^1^http://www.fmrib.ox.ac.uk/fsl

^2^http://afni.nimh.nih.gov/

^3^http://www.fmrib.ox.ac.uk/analysis/research/feat/
